# Area-Level Socioeconomic Characteristics, Prevalence and Trajectories of Cardiometabolic Risk

**DOI:** 10.3390/ijerph110100830

**Published:** 2014-01-08

**Authors:** Anh D. Ngo, Catherine Paquet, Natasha J. Howard, Neil T. Coffee, Anne W. Taylor, Robert J. Adams, Mark Daniel

**Affiliations:** 1 Clinical and Population Perinatal Research, Kolling Institute of Medical Research, University of Sydney at Royal North Shore Hospital, St Leonards, New South Wales, NSW 2065, Australia; 2 Social Epidemiology and Evaluation Research Group, School of Population Health and Sansom Institute for Health Research, University of South Australia, Adelaide, SA 5001, Australia; E-Mails: Catherine.Paquet@unisa.edu.au (C.P.); Natasha.Howard@unisa.edu.au (N.J.H.); Neil.Coffee@unisa.edu.au (N.T.C.); mark.daniel@unisa.edu.au (M.D.); 3 Research Centre of the Douglas Mental Health University Institute, Verdun, QC H4H 1R2, Canada; 4 Population Research and Outcome Studies, Discipline of Medicine, The University of Adelaide, Adelaide, SA 5005, Australia; E-Mail: Anne.Taylor@adelaide.edu.au; 5 The Health Observatory, Discipline of Medicine, The University of Adelaide, Adelaide, SA 5005, Australia; E-Mail: Robert.adams@adelaide.edu.au; 6 Department of Medicine, The University of Melbourne, St Vincent’s Hospital, Melbourne, VIC 3065, Australia

**Keywords:** metabolic syndrome, trajectories, socioeconomic position, income, education, cohort study, residence characteristics

## Abstract

This study examines the relationships between area-level socioeconomic position (SEP) and the prevalence and trajectories of metabolic syndrome (MetS) and the count of its constituents (*i.e.*, disturbed glucose and insulin metabolism, abdominal obesity, dyslipidemia, and hypertension). A cohort of 4,056 men and women aged 18+ living in Adelaide, Australia was established in 2000–2003. MetS was ascertained at baseline, four and eight years via clinical examinations. Baseline area-level median household income, percentage of residents with a high school education, and unemployment rate were derived from the 2001 population Census. Three-level random-intercepts logistic and Poisson regression models were performed to estimate the standardized odds ratio (SOR), prevalence risk ratio (SRR), ratio of SORs/SRRs, and (95% confidence interval (CI)). Interaction between area- and individual-level SEP variables was also tested. The odds of having MetS and the count of its constituents increased over time. This increase did not vary according to baseline area-level SEP (ratios of SORs/SRRs ≈ 1; *p* ≥ 0.42). However, at baseline, after adjustment for individual SEP and health behaviours, median household income (inversely) and unemployment rate (positively) were significantly associated with MetS prevalence (SOR (95%CI) = 0.76 (0.63–0.90), and 1.48 (1.26–1.74), respectively), and the count of its constituents (SRR (95%CI) = 0.96 (0.93–0.99), and 1.06 (1.04–1.09), respectively). The inverse association with area-level education was statistically significant only in participants with less than post high school education (SOR (95%CI) = 0.58 (0.45–0.73), and SRR (95%CI) = 0.91 (0.88–0.94)). Area-level SEP does not predict an elevated trajectory to developing MetS or an elevated count of its constituents. However, at baseline, area-level SEP was inversely associated with prevalence of MetS and the count of its constituents, with the association of area-level education being modified by individual-level education. Population-level interventions for communities defined by area-level socioeconomic disadvantage are needed to reduce cardiometabolic risks.

## 1. Introduction

Cardiometabolic disease (*i.e.*, cardiovascular disease and type 2 diabetes) is a leading cause of mortality and morbidity, and accounts for a significant burden of disease worldwide [1]. A range of socioeconomic, behavioural, and biological risk factors shape the distribution and development of cardiometabolic disease. Metabolic syndrome (MetS) is a cluster of disturbed glucose and insulin metabolism, obesity or abdominal adiposity, dyslipidemia, and hypertension. Studies have shown that cardiometabolic disorders tend to cluster [2] and people with multiple disorders who meet criteria for MetS have an increased risk for cardiometabolic disease morbidity [3,4], cardiovascular mortality, and all-cause mortality [5]. In addition there is a continuum of elevated risk as the number of MetS components rises. Studies conducted in the United States (US) indicate that cardiovascular disease incidence, cardiovascular and all-cause mortality rise incrementally as the number of MetS components increases [4,6]. 

Ample evidence links lower individual-level SEP with a higher prevalence and incidence of elevated cardiometabolic risk factors including MetS [7,8,9,10,11,12,13,14,15,16,17,18,19,20], its clinical components [21,22,23,24,25,26,27,28,29,30], and overweight and obesity [31]. Additionally, area-level socioeconomic environments are proposed to influence cardiometabolic health outcomes through materials and resources that shape individual behaviours and biological responses [32]. A growing number of studies have demonstrated an inverse association between area-level SEP and prevalence of MetS [33,34,35,36] and its components, including hypertension [37,38,39,40], overweight and obesity [38,41,42], and the incidence of hypertension [43], diabetes [44,45], and obesity [46]. Area-level studies also indicate that low SEP individuals can be more vulnerable to the adverse effects of area-level socioeconomic disadvantage on cardiometabolic outcomes [47]. 

Few longitudinal studies have examined the trajectories of change in cardiometablic risk factors as they vary according to area-level SEP. Results of available studies are inconsistent. Three US-based longitudinal studies have reported cross-sectional associations between area-level SEP and body mass index (BMI) at baseline [48,49,50], but only one of these studies also provided evidence for longitudinal associations in the analysis. Analyses indicated that women, but not men, living in the most deprived neighbourhoods had a greater increase in BMI over a 13-year follow-up period [50]. In one study [49], it was noted that persons who already had high BMI at baseline may be unlikely to increase even more over time, leading to the lack of a longitudinal relationship. In a different study, 5-year change in systolic blood pressure did not vary by neighbourhood SEP score, while 5-year increases in diastolic blood pressure were greater in participants living in low SEP areas [51]. The above studies used a composite measure to evaluate area-level SEP. While this method can capture multifaceted nature of SEP, it does not allow for disentangling the independent effects of specific features of area-level SEP. Furthermore, no longitudinal studies have considered trajectories of other cardiometabolic risk markers, such as impaired glycaemia, dyslipidemia and MetS. 

Individuals can move “in” and “out” of MetS in a given time period, depending on changes in levels of its clinical components. Similarly, the count of individual risk factors that constitute MetS can change over time. Therefore, in assessing how area-level SEP impacts metabolic abnormalities, it is important to evaluate trajectories of change in both MetS and the count of its constituents. This longitudinal study aimed to advance knowledge on the influences of socioeconomic environments on cardiometabolic risk by examining the prevalence and trajectories of MetS and the count of its constituents as clusters of risks and stronger risk markers of cardiometabolic disease and their associations with area-level SEP factors. Our primary hypothesis was that living in areas with a higher level of socioeconomic disadvantage would be associated with a greater increase in the risk of MetS as well as a greater increase in the count of its components over time (hypothesis 1—longitudinal associations). Additionally, given that socioeconomic gradient in the risk of MetS and its components could have been present at the onset of the study, we also tested whether living in areas with a higher level of socioeconomic disadvantage would be associated with a higher prevalence of MetS and a greater count of its components at baseline (hypothesis 2—cross-sectional associations). 

## 2. Research Design and Methods

### 2.1. Study Population

This study was conducted as part of the Place and Metabolic Syndrome (PAMS) project, a research initiative that aims to evaluate the relationships between local-area social and built environmental factors and cardiometabolic health. The project draws on the North West Adelaide Health Study (NWAHS), a population-based prospective cohort study designed to provide longitudinal self-reported and clinically measured data on a number of chronic health conditions and disease risk factors [52]. A total of 4,056 men and women aged 18 years or over were recruited between 2000 and 2003 through a random selection of households that, at time of baseline sampling, had a telephone with the corresponding number listed in the Electronic White Pages directory. Participants were subsequently re-surveyed and re-examined during two additional waves of data collection: Wave 2 (2005–2006) and Wave 3 (2008–2010). At baseline and each subsequent wave, participants with a valid street residential address were assigned a georeference, representing the longitude and latitude of their residential location. A more complete description of the cohort, data collection and measurements has been published previously [52]. Ethics approval for the PAMS project was obtained from three Human Research Ethics Committees: the University of South Australia, Central Northern Adelaide Health Service, and the South Australian Department of Health and Ageing. 

### 2.2. Measurements

#### 2.2.1. Cardiometabolic Risk Measures

MetS was defined, using the International Diabetes Foundation criteria [53], as abdominal obesity (waist circumference ≥ 94 cm for Europid men and ≥90 cm for non-Europid men, and ≥80 cm for Europid and non-Europid women) plus any two or more of the following four metabolic abnormalities: Hypertriglyceridemia (≥1.7 mmol/L) or specific treatment for this lipid abnormality; low high-density lipoprotein (HDL) cholesterol level (≤1.3 mmol in women, ≤1.0 mmol in men) or specific treatment for this lipid abnormality; elevated blood pressure (≥130/85 mm Hg) or treatment of previously diagnosed hypertension; and hyperglycemia (fasting plasma glucose > 6.1 mmol/L) or previously diagnosed type 2 diabetes. The count of MetS components is the number of individual risk factors that constitute MetS, ranging from zero to five. For the sake of brevity, this variable is called MetS component count throughout the paper. 

#### 2.2.2. Area-level Socioeconomic Position

The spatial unit used to define area-level SEP was the 2001 State Suburb, an Australian Bureau of Statistics (ABS) Census Geographic Unit [54] that defines administrative boundaries (*i.e.*, suburbs) within urban areas. The State Suburbs are a derived spatial unit corresponding to recognised localities within Australian States. State Suburbs located in the study region have a median population of 2,027 persons and a median size of 1.47 km^2^ [54]. This spatial unit was selected based on prior analyses suggesting that State Suburbs were associated with the greatest level of clustering for a range of continuous cardio-metabolic risk factors among five different spatial units being considered. 

The study region consisted of 154 State Suburbs at baseline. A geographic information system (GIS) was utilised to assign each participant to a State Suburb, based on their Wave 1 georeference. Within each spatial unit, three area-level SEP measures were extracted for analysis from the 2001 Census of Population and Housing [55]: percentage of residents aged 18 years or older with a high school education or more, median household weekly income (in Australian Dollars (AUD)), and the unemployment rate defined as the proportion of residents aged 15 or older within each State Suburb who were unemployed. 

#### 2.2.3. Covariates

Individual-level SEP was measured at baseline in terms of self-reported educational attainment (having a post-high school education or not; counterpart to area-level education), annual household income classified as low (<20,000 AUD), middle (20,000–60,000 AUD), or high (>60,000 AUD) (counterpart to area-level income), and employment status (counterpart to the area-level unemployment rate). Employment status was categorized as either “currently in the workforce” (*i.e.*, full-time or part-time/casual employment) *versus* “currently not in the workforce” (unemployed, student, homemaker, retired, or other). Health behaviours were self-reported using questions previously employed in the ABS National Health Survey [56]. Smoking status was classified as either current, former, or non-smoker. Alcohol consumption was defined as either at risk and not at risk drinkers, based on national guidelines [57]. Male participants were coded “at risk” if they consumed more than six standard drinks on any one day, an average of more than four drinks per day, or more than 28 standard drinks over a week. Female participants were coded “at risk” if they consumed more than four standard drinks on any one day, an average of more than two drinks per day, or more than 14 standard drinks over a week.

Physical activity was expressed as Metabolic Equivalence Tasks (METs) in hours per week [58] derived from the total amount and intensity of physical activity carried out for sport, recreation or fitness within the two weeks preceding survey completion. Participants were assigned to either sedentary, low, moderate, or high classifications if they achieved ≤100 METs, 100–≤ 1,600 METs, 1,600–≤ 3,200 METs, and >3,200 METs, respectively. 

### 2.3. Statistical Analysis

We fitted three-level random intercepts logistic and Poisson regression models to estimate the relationships between each measure of area-level SEP (treated as time-invariant variable) and the trajectories of MetS and its component counts. The multi-level statistical approach allows for the inclusion of participants with one missing clinical visit and the evaluation of both cross-sectional (at baseline) and longitudinal relationships. In these models, measurement occasion (level 1) was nested within participants (level 2) who were nested within State Suburb (level 3). The intercept was treated as random for levels 1 and 2 (representing baseline MetS, its component count, and between-subject variation); slope was specified as random for level 1 only (representing the linear change in each outcome by year and participant’s departure from the overall slope). All other parameters were defined as fixed.

All analyses were performed with Stata version 12.0 [56]. The Gllamm command was used to fit three-level Poisson regression models with a robust option to account for over-dispersion [59]. The Xmetlogit command was used to fit three-level logistic regression models. The inclusion of a random term for the slope of time led to convergence problems for some logistic regression models, most likely due to the small number of repeated measurements as reported previously [60]. For models that converged, the exclusion of a random term for the slope of time did not produce a fundamental change in the estimate of both cross-sectional and longitudinal relationships between SEP measures and MetS. Logistic regression models were therefore estimated using only a fixed term for the slope of time. 

Each area-level SEP measure was tested in four sequential statistical models. First model included time since the first clinical visit (expressed in years), an area-level SEP measure, and their two-way interaction term to estimate the following parameters: (i) odds ratio (OR) and risk ratio (RR) representing changes in outcomes for every year elapsed; (ii) standardised prevalence OR (SOR) and standardised prevalence RR (SRR) respectively, representing the associations between area-level SEP and MetS and its component count at baseline (cross-sectional relationships); and (iii) ratio of SORs/SRRs representing change in outcomes over time in relation to area-level SEP (longitudinal relationships). This model also included age (expressed in years) at baseline and gender, a two-way interaction term between each area-level SEP measure and gender, and a three way interaction term between each area-level SEP measure, gender, and time (Model 1). To test whether area-level SEP had independent associations with MetS and its component count, above and beyond associations with individual-level SEP and health behaviours, subsequent nested models included the counterpart individual-level SEP plus a two-way interaction term between area- and individual-level SEP, and a three-way interaction term between area- and individual-level SEP, and time (Model 2), and behavioural variables (*i.e.*, smoking, alcohol consumption, and physical activity) (Model 3). Last, to test the relative importance of specific expressions of area-level SEP in predicting the prevalence and trajectories of outcomes, all other area- and individual-level SEP variables were added (Model 4). Non-significant interaction terms were removed from the model. For significant interaction terms, stratified analyses were performed to depict the nature of the relationship. 

Participants who moved to another State Suburb prior to the second assessment (n = 599 (17%)) were excluded from analysis. This exclusion ensures that all participants from the same residential area at baseline had been exposed to the same environment for an extended period of time prior to the ascertainment of outcomes. This approach has been recommended to yield a more accurate assessment of exposure to area-level characteristics than would analyses that included participants who moved [61]. It has been used in a number of previous longitudinal studies linking area-level factors to individual cardiometabolic health outcomes [47,61,62,63]. 

Retention rates of the cohort at Wave 2 and Wave 3 were 79% and 62%, respectively. To explore the possible impact of loss to follow up on our estimates, we examined the distribution of age, gender, and individual-level education, income, and employment status, and area-level SEP characteristics in relation to the number of completed clinical visits.

## 3. Results and Discussion

### 3.1. Descriptive Statistics

The sample available for the current analysis contained 2,619 participants (82.5% of 3,175 Wave 1—Wave 2 non-movers) who attended at least two clinical examinations. The median number of participants included per State Suburb was 13, ranging from 1 to 80. The median follow-up time was 3.9 years, varying from 1.8 to 6.2 years between Wave 1 and Wave 2 (n = 2,508), and 8.2 years, varying from 5.5 to 10.3 between Wave 1 and Wave 3 (n = 1,984). Descriptive information on study participants is presented in Table 1.

MetS prevalence rose slightly from 36.2% at Wave 1 to 37.8% at Wave 2 and to 39.7% at Wave 3. Sixteen percent or more of participants who were free of MetS at Wave 1 or Wave 2 developed MetS at a subsequent wave (MetS incidence). Twenty two percent or more of participants who had MetS at Wave 1 or Wave 2 reversed this status at a subsequent wave (MetS reversal). The average number of MetS components increased from 2.06 at Wave 1 to 2.11 at Wave 2 and to 2.16 at Wave 3 (Table 1). 

**Table 1:** Baseline characteristics of participants included in analyses (n = 2,619).

Characteristic	n/median/mean	Percentage/range
**Age (years)**	50	18–90
**Gender**		
Male	1,253	47.8
Female	1,336	52.2
**Household income**		
Low (<20,000 AUD)	802	30.6
Middle (20,00–60,00 AUD)	1,270	48.5
High (>60,000 AUD)	547	20.9
**Individual education**		
Post-secondary education	1,734	54.6
No post-secondary education	1,441	45.4
**Individual employment status**		
Currently in the workforce	1,387	53
Not currently in the workforce	1,232	47
**Physical Activity**		
Sedentary	775	29.6
Low exercise	919	35.1
Moderate exercise	668	25.5
High exercise	257	9.8
**Smoking**		
Current	417	16.3
Former	980	37.4
Never	1,212	46.3
**Drinking**		
Not at risk	1,954	74.6
At risk	665	25.4
**Area-level SEP**		
Area-level high school education (%)	71.5	49.7–88.5
Area-level weekly income (AUD)	621	361–1,323
Are-level unemployment rate (%)	6.3	0–14.8
**MetS prevalence**		
Wave 1 (n = 3,175)	1,150	36.2
Wave 2 (n = 2,492)	942	37.8
Wave 3 (n = 1963)	780	39.7
**MetS incidence**		
Between Wave 1–Wave 2		16.0
Between Wave 2–Wave 3		20.0
Between Wave 1–Wave 3		17.5
**MetS reversal**		
Between Wave 1–Wave 2		25.7
Between Wave 2–Wave 3		25.1
Between Wave 1–Wave 3		22.2
**Mean number of MetS components**		
Wave 1	2.06	
Wave 2	2.11	
Wave 3	2.16	

As shown in Table 2, there was evidence that the number of clinic visits from which MetS status and its component count were ascertained varied according to measures of both individual- and area-level SEP. The proportion of participants with high income, currently in the workforce, and living in more advantaged areas (*i.e.*, areas with higher percentage of high school graduates, higher household income, or lower unemployment rate) was higher for participants who completed all three visits compared with those who completed one or two visits.

**Table 2:** Distribution of baseline variables by the number of clinical visits for which MetS status was ascertained.

	One visit	Two visits	Three visits
	(n = 542)	(n = 760)	(n = 1,873)
**Mean age at baseline (SD)**	50.3 (17.7)	50.7(16.7)	50.7(16.0)
**Gender (%male)**	48.0	48.1	47.8
**Education (% with a high school education)**	53.2	50.2	56.8
**Household income (%)**			
Low (<20,000 AUD)	38.8	38.2	27.8
Middle (20,000–60,000 AUD)	43.7	45.4	49.7
High (>60,000 AUD)	17.5	16.5	22.5
**Employment status (%)**			
Currently in the workforce	47.6	42.9	56.7
Not currently in the workforce	52.4	57.1	43.3
**Area-level SEP**			
Median % of high school graduates (25th, 75th)	70.6 (66.0, 77.4)	70.6 (65.4, 77.4)	72.3 (67.1, 77.6)
Median of median household weekly income (25th, 75th)	653 (515, 731)	648 (509, 731)	661 (537, 744)
Median unemployment rate (25th, 75th)	6.4 (4.9, 7.7)	6.3 (4.9, 8.0)	6.0 (4.8, 7.2)

### 3.2. Relationships between Area-Level SEP and Baseline Prevalence and Trajectories of MetS and its Component Count

In examining the interaction of area-level SEP with gender or individual-level SEP, only two-way interaction term between area- and individual-level education was statistically significant (*p* ≤ 0.025). Therefore, an analysis stratified according to individual-level education was then performed (Table 3 and Table 4). 

Consistent with hypothesis 2, at baseline, participants living in areas with higher median weekly household income or lower unemployment rate, independent of their own household income or employment status, had a statistically lower odds of having MetS and a lower MetS component count. Specifically, the odds of having MetS was lowered by 24% with the average component count reduced by 4% for each sd increase in median household weekly income, and the odds of having MetS grew by 48% with the average component count increased by 7% for each sd increase in the unemployment rate (Model 2). The associations persisted in the subsequent analysis accounting for health behaviours (Model 3), except for the association between area-unemployment and baseline prevalent MetS where a slight reduction in OR was observed (from 1.53 to 1.48). 

The results indicated that the odds of having MetS and its component count tended to increase over time, independent of individual- and area-level SEP factors and health behaviours (for every year elapsed, OR or RR > 1.0). However, the analysis indicated no statistically significant interaction between any measure of area-level SEP and time since the first clinic visit (*p ≥* 0.42), indicating that such increase did not vary according to baseline area-level SEP. These results do not support hypothesis 1. 

Analyses stratified according to individual-level education indicated that, at baseline, the association between area-level education and MetS or its component count was statistically significant only amongst participants with less than a post-high school education. A one sd increase in the proportion of high school graduates corresponded to a 42 % reduction in the odds of having MetS, and a 9 % decrease in the average count of MetS components in this group, even after accounting for health behaviours (Model 3). After controlling for all other individual- and area-level SEP variables (Model 4), however, the association of area-level income and education with each outcome did not remain statistically significant (*p* ≥ 0.54).

**Table 3:** Associations between area-level SEP and baseline prevalence and trajectory of MetS.

Characteristic	Model 1	Model 2	Model 3	Model 4
	OR/SOR (95%CI)	OR/SOR (95%CI)	OR/SOR (95%CI)	OR/SOR (95%CI)
**INCOME**				
Median household weekly income (for 1sd increase)	0.62 (0.51–0.74)^ b^	0.75 (0.63–0.90)^ b^	0.76 (0.63–0.90)^ b^	1.09 (0.74–1.61)
Time	1.05 (1.03–1.08)^ b^	1.05 (1.03–1.08)^ b^	1.05 (1.03–1.08)^ b^	1.06 (1.03–1.08)^ b^
Time × Area income	1.00 (0.97–1.03)	1.00 (0.97–1.03)	1.00 (0.97–1.03)	1.00 (0.97–1.03)
**EMPLOYMENT**				
Are-level unemployment rate (for 1 sd increase)	1.66 (1.42–1.96)^ b^	1.53 (1.30–1.80)^ b^	1.48 (1.26–1.74)^ b^	1.46 (1.15–1.87)^ b^
Time	1.05(1.03–1.08)^ b^	1.06(1.03–1.08)^ b^	1.06 (1.03–1.08)^ b^	1.06 (1.03–1.08)^ a^
Time × Area unemployment rate	0.99 (0.97–1.02)	0.99 (0.97–1.01)	0.99 (0.97–1.01)	0.99 (0.97–1.01)
**NO POST-HIGH SCHOOL EDUCATION**				
Proportion of high school graduates (for 1 sd increase)	0.55 (0.43–0.70)^ b^		0.58 (0.45–0.73)^ b^	0.88 (0.57–1.34)
Time	1.07 (1.03–1.10)^ b^		1.06 (1.02–1.10)^ b^	1.07 (1.03–1.11)^ b^
Time × Area. % high school graduates	1.02 (0.98–1.05)		1.01 (0.98–1.05)	1.01 (0.98–1.05)
**POST-HIGH SCHOOL EDUCATION**				
Proportion of high school graduates (for 1 sd increase)	0.84 (0.67–1.06)		0.88 (0.70–1.10)	1.00 (0.69–1.45)
Time	1.05 (1.01–1.08)^ b^		1.05 (1.01–1.08)^ b^	1.05 (1.02–1.08)^ b^
Time × Area. % high school graduates	0.99 (0.96–1.03)		0.99 (0.96–1.02)	0.99 (0.96–1.02)

Notes: Model 1: Testing each SEP variable separately, adjusted for age, gender, time period; Model 2: Testing each SEP variable separately, adjusted for age, gender, time period, and the individual-level counterpart SEP variable; Model 3: Testing each SEP variable separately, adjusted for all variables in model 2 plus physical activity, smoking habit, and alcohol consumption; Model 4: Including all SEP variables and covariates. ^a^ *p* < 0.05; ^b^ *p* < 0.01.

**Table 4:** Associations between area-level SEP and baseline prevalence and trajectory of MetS component count.

Characteristic	Model 1	Model 2	Model 3	Model 4
	RR/SRR (95%CI)	RR/SRR (95%CI)	RR/SRR (95%CI)	RR/SRR (95%CI)
**INCOME**				
Median household weekly income (for 1 sd increase)	0.92 (0.89–0.96)^ b^	0.96 (0.93–0.99)^ a^	0.96 (0.93–0.99)^ b^	1.04 (0.98–1.11)
Time	1.006 (1.004–1.010)^ b^	1.008 (1.003–1.013)^ a^	1.007 (1.002–1.012)^ a^	1.008 (1.003–1.013)^ b^
Time × median household income	1.00 (0.99–1.01)	1.00 (0.99–1.01)	1.00 (0.99–1.01)	1.00 (0.99–1.01)
**EMPLOYMENT**				
Area-level unemployment rate (for sd increase)	1.09 (1.06–1.12)^ b^	1.07 (1.05–1.10)^ b^	1.06 (1.04–1.09)	**1.07 (1.03–1.11)**^ b^
Time	1.007 (1.002–1.012)^ b^	1.008 (1.003–1.013)^ b^	1.008 (1.003–1.013)^ b^	1.008 (1.003–1.013)^ b^
Time × Area unemployment rate	1.00 (0.99–1.01)	1.00 (0.99–1.01)	1.00 (0.99–1.01)	1.00 (0.99–1.01)
**NO POST-HIGH SCHOOL EDUCATION**				
% High school graduates (for 1sd increase)	0.92 (0.89–0.95)^ b^		0.91 (0.88–0.94)^ b^	0.99 (0.93–1.05)
Time	1.006 (1.000–1.014)		1.006 (1.000–1.014)	1.008 (1.001–1.015) ^a^
Time × Area. % high school graduates	1.00 (0.99–1.01)		1.00 (0.99–1.01)	1.00 (0.99–1.01)
**POST-HIGH SCHOOL EDUCATION**				
% High school graduates (for 1 sd increase)	0.97 (0.93–1.01)		0.96 (0.92–1.00)	0.99 (0.92–1.05)
Time	1.008 (1.001–1.015)^ b^		1.008 (1.001–1.015)^ b^	1.009 (0.992–1.006)^ b^
Time × Area. % high school graduates	1.00 (0.99–1.01)		1.00 (0.99–1.01)	1.00 (0.99–1.01)

Notes: Model 1: Testing each SEP variable separately, adjusted for age, gender, time period; Model 2: Testing each SEP variable separately, adjusted for age, gender, time period, and the individual-level counterpart variable; Model 3: Testing each SEP variable separately, adjusted for all variables in model 2 plus physical activity, smoking habit, and alcohol consumption; Model 4: Including all SEP variables and covariates. ^a^ *p* < 0.05; ^b^ *p* < 0.01.

It is noted that in all statistical models, there were statistically significant associations between individual SEP, smoking, and physical activity with MetS prevalence and its component count at baseline. Specifically, participants without a post high school education, earning a lower income, and not being in the workforce, being a former smoker, and having a lower level of physical activity were more likely than others to have MetS and higher number of its components (data not shown). Alcohol consumption was not consistently associated with these two outcomes. 

## 4. Discussion

This study is the first to examine the capacity of area-level SEP factors to predict actual trajectories of change in cardiometabolic risk expressed as MetS and the count of its components. Our results indicate that area-level SEP does not predict differences in trajectories of MetS or its component count over time. The absence of a longitudinal relationship in these analyses is consistent with earlier longitudinal studies that have documented cross-sectional (at baseline) but not longitudinal relationships between a composite index of area-level SEP and trajectories of BMI and systolic blood pressure [48,49,50,51]. On the other hand, our results contradict a different study that documented a longitudinal relationship between area-level SEP and BMI in women over 13 years of follow up [45]. The use of different measures to rank area-level SEP, consideration of different cardiometabolic risk outcomes, and different follow up durations may account for these inconsistent findings between studies.

In contrast to the absence of a longitudinal relationship, at baseline, participants living in more socioeconomically advantaged areas (high median household weekly income or low unemployment rate) exhibited, independent of their own income, employment status, or health behaviour, a lesser odds of having MetS and a lesser count of its components compared to participants residing in lower SEP areas. An inverse cross-sectional association between area-level education and all outcomes, however, was only observed for participants without at least a high school education. The lack of differences in trajectories of MetS and its component count according to area-level SEP indicates that the socioeconomic gradient in cardiometabolic risk established at baseline would have persisted over the study period.

It has been proposed that area-level socioeconomic conditions both reflect and shape the social and built environments of one’s residential area and thus subsequently affect cardiometabolic risk [64]. Presence of a cross-sectional association between area-level SEP and cardiometabolic risk at baseline suggests that this gradient may have occurred prior to the initial clinical examination. Those area-level SEP indicators measured at baseline possibly also reflect the pattern of prior exposure to features of the residential environments (e.g., walkability, safety, social norms) that may have shaped local residents’ cardiometabolic risk factors (e.g., physical inactivity, unhealthy diet, smoking, environmental stressors). Assuming that people did not move to a new residential area or had lived in a residential area with a similar SEP since their childhood or adolescence, this pattern may have evolved from the developmental period when environmental factors, through area-level SEP, most strongly shape the evolution of individual lifestyle and behaviour, and thus influence the development of metabolic abnormalities and MetS. There is also a possibility that persons with worse cardiometabolic health outcomes may be more likely to reside in low SEP areas and more healthy people may be more likely to migrate to areas with better socioeconomic conditions. Accordingly, the socioeconomic gradient in cardiometabolic risk observed at baseline may have existed before the onset of the study when the SEP patterns in cohort participants present. 

The presence of an interaction between individual- and area-level education at baseline suggests that the adverse effects of lower area-level education, a marker of a less healthful living environment (e.g., with lesser neighbourhood walkability [65], lesser availability of healthy foods [65,66], or higher prevalence of chronic stressors [67]) were only present amongst individuals with lesser education. It has been postulated that individual-level education can contribute to a relationship between area-level education and individual cardiometabolic outcomes by shaping one’s perceptions of the neighbourhood environment, where these perceptions then influence the adoption and maintenance of health behaviours as well as capacity to cope with stressors [56]. It is also likely that higher individual education renders greater health literacy, leading to rapid dissemination of healthy behaviours (e.g., physical activity, healthy diet) through the formation of social norms among residents within a neighbhourhhood. Greater health literacy can also generate more healthful responses to the negative effects of adverse neighbourhood environments. This hypothesis regarding underlying mechanisms might help explain why individual-level education, in this analysis, modified the relationships between area-level education and cardiometabolic risk outcomes observed at the study onset.

The study has important strengths. First, it is the first study using a longitudinal design to evaluate the trajectories of MetS and the count of its clinical components in relation to area-level SEP characteristics. In focusing on several related cardiometabolic risks, the study findings would be more robust compared to previous studies that considered only a single risk marker. Second, the study overcomes shortcomings associated with the use of composite measures of area-level SEP, unraveling relationships to assess the specific, independent effects of key characteristics (*i.e.*, area-level education, income, and unemployment rate) on the change in trajectories of cardiometabolic risks. Third, compared to conventional analytical approaches to longitudinal data (e.g., modelling the subsequent outcomes as a function of outcomes ascertained in previous measurements), the use of multilevel analytical modelling allows for the optimal use of available data (*i.e.*, inclusion of participants with one missing follow-up clinical examination who would have otherwise been excluded from the analysis), and the results are therefore less susceptible to bias due to loss to follow-up. 

Study limitations should be acknowledged. First, as our analysis indicated, loss to follow-up was more likely for participants having a lower SEP or living in lower SEP areas. This differential loss to follow-up could have diminished the variations in trajectory of cardiometabolic risks in relation to area-level SEP. To evaluate the effects of differential loss follow-up, we conducted a sensitivity analysis, replacing missing data based on the following assumptions: participants who were lost to follow-up at Wave 3 all developed five MetS components if they were from low-SEP areas that had the median household weekly income or the percentage of high school graduates belong to either: (i) the lowest quartile, (ii) two lowest quartiles, or (iii) three lowest quartiles, or the unemployment rate are within either: (i) the highest quartile, (ii) two highest quartiles, or (iii) three highest quartiles, while the remaining participants (from higher SEP areas) who were lost to follow-up at Wave 3 did not develop any MetS components. In all scenarios, our analysis did not find a fundamental change in the point estimate and 95%CI for SORs/SRRs and ratios of SORs/SRRs to either direction from unity. Such consistent findings give confidence that the absence of trajectory differences according to baseline area-level SEP was reliable despite differential loss follow-up. 

Second, it is possible that gentrification or changes in the composition of local residents due to movements and flows of the population occurred during follow-up in some areas. These changes can make baseline SEP indicators non-representative of the area environment to which participants were exposed during the follow-up period. Third, as State Suburbs were used as proxies for residential areas, the findings are potentially susceptible to the modifiable area unit problem [68] and reported associations may be reflective of the underlying spatial properties (*i.e.*, size variations, the level of aggregation and the configuration of spatial units). Finally, the use of area-level SEP measures to characterise residential areas may not adequately capture other features of the living environment relevant to cardiometabolic health, such as area-level walkability, safety, and the availability of healthy foods.

## 5. Conclusions

This study extends and confirms prior work that examined the link between area-level SEP factors and trajectories of change in cardiometabolic risk by modelling changes in the risk for MetS and its component count. Together with past studies, the findings raise important questions regarding the relationship between area-level SEP factors and trajectories of change in cardiometabolic risk in adult populations. From adulthood, area-level SEP appears not to be a causal determinant of the evolution of cardiometabolic risk over time. However, our observation of an inverse relationship at baseline that would have persisted over the study period signifies the need for population level interventions for communities defined by area-level socioeconomic disadvantage to reduce prevalence of cardiometabolic risks, such interventions being particularly important for less individually educated residents.

Our study limitations would suggest that in future studies, regular updates of area-level SEP indicators be used to account for possible changes in area-level SEP during the follow-up period. Future studies should also track person-level trajectories of MetS and its clinical components from earlier life stages, prior to the emergence of socioeconomic gradients. Furthermore, future analysis should take into account the length of residence in a particular area and assess the role of area-level SEP factors in conjunction with other environmental factors relevant to cardiometabolic health, such as neighbourhood walkability, safety, social cohesion, and health and social service availability in the trajectories of cardiometabolic risk.

## Abbreviations

ABS: Australian Bureau of Statistics
AUD: Australian Dollar
BMI: Body Mass Index
CATI: Computer-Assisted Interview
CI: Confidence Interval
HDL: High-Density Lipoprotein
MetS: Metabolic Syndrome
NWAHS: North West Adelaide Health Study
SOR: Standardised Odds Ratio
PAMS: Place and Metabolic Syndrome
SRR: Standardised Prevalence Risk Ratio
SA: South Australia
SEP: Socioeconomic Position
SD: Standard Deviation
